# Rapid discrimination between clinical *Clostridioides difficile* infection and colonization by quantitative detection of TcdB toxin using a real-time cell analysis system

**DOI:** 10.3389/fmicb.2024.1348892

**Published:** 2024-01-23

**Authors:** Yuhang Shen, Shan Lin, Peijun You, Yu Chen, Yun Luo, Xiaojun Song, Yunbo Chen, Dazhi Jin

**Affiliations:** ^1^School of Laboratory Medicine, Hangzhou Medical College, Hangzhou, China; ^2^Key Laboratory of Biomarkers and In Vitro Diagnosis Translation of Zhejiang Province, Hangzhou, China; ^3^Institute of Ageing Research, School of Basic Medical Sciences, Hangzhou Normal University, Hangzhou, China; ^4^TEDA Institute of Biological Sciences and Biotechnology, Nankai University, Tianjin, China; ^5^School of Biotechnology and Biomolecular Sciences, University of New South Wales, Sydney, NSW, Australia; ^6^Laboratory Medicine Center, Department of Clinical Laboratory, Zhejiang Provincial People’s Hospital, Hangzhou Medical College, Hangzhou, China; ^7^State Key Laboratory for Diagnosis and Treatment of Infectious Diseases, The First Affiliated Hospital, School of Medicine, Zhejiang University, Hangzhou, China

**Keywords:** *Clostridioides difficile*, infection, colonization, real-time cell analysis, TcdB

## Abstract

**Objectives:**

It is important to accurately discriminate between clinical *Clostridioides difficile* infection (CDI) and colonization (CDC) for effective antimicrobial treatment.

**Methods:**

In this study, 37 stool samples were collected from 17 CDC and 20 CDI cases, and each sample were tested in parallel through the real-time cell analysis (RTCA) system, real-time PCR assay (PCR), and enzyme-linked immunosorbent assay (ELISA).

**Results:**

RTCA-measured functional and toxical *C. difficile* toxin B (TcdB) concentrations in the CDI group (302.58 ± 119.15 ng/mL) were significantly higher than those in the CDC group (18.15 ± 11.81 ng/mL) (*p* = 0.0008). Conversely, ELISA results revealed no significant disparities in TcdB concentrations between the CDC (26.21 ± 3.57 ng/mL) and the CDI group (17.07 ± 3.10 ng/mL) (*p* = 0.064). PCR results indicated no significant differences in *tcdB* gene copies between the CDC (774.54 ± 357.89 copies/μL) and the CDI group (4,667.69 ± 3,069.87 copies/μL) (*p* = 0.407). Additionally, the functional and toxical TcdB concentrations secreted from *C. difficile* isolates were measured by the RTCA. The results from the CDC (490.00 ± 133.29 ng/mL) and the CDI group (439.82 ± 114.66 ng/mL) showed no significant difference (*p* = 0.448). Notably, RTCA-measured functional and toxical TcdB concentration was significantly decreased when mixed with pooled CDC samples supernatant (*p* = 0.030).

**Conclusion:**

This study explored the novel application of the RTCA assay in effectively discerning clinical CDI from CDC cases.

## 1 Introduction

*Clostridioides difficile*, a major contributor to antimicrobial-associated infectious diarrhea, is prevalent in both human and animal intestines and the environment (Czepiel et al., 2019). *C. difficile* infection (CDI) presents with a spectrum of clinical symptoms, from mild diarrhea and fever to severe conditions such as toxic megacolon, intestinal perforation, and, in extreme cases, septic shock and death (Rupnik et al., 2009). The virulence of *C. difficile* is significantly influenced by Toxin B (TcdB) (Carter et al., 2012), with clinical symptom severity directly correlating to TcdB concentrations in fecal samples (Ryder et al., 2010). Despite extensive documentation of *C. difficile* colonization (CDC) with toxigenic strains, prevalence rates vary widely from 4 to 71% across different populations (Crobach et al., 2018). Studies emphasize the potential role of CDC as an infection reservoir, contributing to healthcare-associated CDI transmission and posing a high risk of progression to CDI in hospitalized patients (Crobach et al., 2018). Thus, a crucial challenge involves differentiating between CDI and CDC during clinical diagnosis for subsequent anti-infective treatment.

A CDI case is defined by a combination of clinical and laboratory results, including the presence of diarrhea with either a positive stool test result for toxigenic *C. difficile* or pseudomembranous colitis. CDC is defined as the presence of toxigenic *C. difficile* in a patient without CDI symptoms (Crobach et al., 2018). Although toxigenic culture (TC) is considered the “gold standard”, it’s impractical for clinical use due to subjectivity and complexity (Huang et al., 2014b). Current clinical laboratory methods, using molecular techniques to rapidly detecting toxin genes and proteins, are difficult to discriminate between CDI and CDC due to inherent limitations (Huang et al., 2014b). Enzyme immunoassays (EIA) swiftly detect Toxin A/B and glutamate dehydrogenase (GDH) with sensitivity and specificity of 50%–90% and 70%–95%, respectively (Mohan et al., 2006). Some molecular assays show promise, but high sensitivity may overdiagnosis toxigenic CDC (Guh et al., 2020). Debate persists on treating patients with positive molecular but negative toxin immunoassay results (Polage et al., 2015).

The real-time cell analysis (RTCA) system has been applied for the quantitative detection of functional and toxical *C. difficile* TcdB in clinical samples (Ryder et al., 2010; Huang et al., 2014a). Our previous studies have demonstrated that the potential of RTCA in determining the severity of clinical CDI before treatment and monitoring therapeutic efficacy by assessing functional and toxical TcdB concentrations (Ryder et al., 2010). However, it remains unclear whether the RTCA can effectively distinguish between CDI and CDC based on measured functional and toxical TcdB concentrations. In this study, we utilized the established RTCA assay to quantitatively measure functional and toxical TcdB concentrations in clinical stool samples obtained from patients with CDI and CDC, respectively. The performance of the RTCA were also assessed in comparison to the ELISA and real-time PCR assays in these two types of samples.

## 2 Materials and methods

### 2.1 Cell lines, TcdB, and antibodies

Four cell lines, HeLa (CRM-CCL-2), HS27 (CRL-1634), U87 (HTB-14) and Caco-2 (HTB-37) were procured from the American Type Culture Collection (ATCC) and cultured adherently in Dulbecco’s Modified Eagle’s Medium (DMEM). The culture medium was supplemented with 1% glutamine (Wuhan Pricella Bi Co., Ltd., Hubei, China), 10% fetal bovine serum (Thermo Fisher Scientific, Inc., Waltham, United States), and 1% penicillin–streptomycin solution (Wuhan Pricella Bio Co., Ltd., Hubei, China) (Ryder et al., 2010). Purified TcdB was prepared in a cold phosphate buffer solution (PBS) in our laboratory, and TcdB neutralization antibodies were acquired from Diagnostic Hybrids Inc. (Athens, United States).

### 2.2 Relative quantification of expression of TcdB-receptor genes

After incubating HeLa, HS27, U87 and Caco-2 cells at 37°C in a cell incubator to induce confluent monolayer formation, total RNAs were extracted using the MiniBEST Universal RNA Extraction Kit (Takara Bio Inc., Shiga, Japan). Subsequently, cDNAs were synthesized with the PrimeScript^™^ RT kit (Takara Bio Inc., Shiga, Japan), and the expression levels of four TcdB-receptor genes (*CSPG4*, *PVRL3*, *FZD1*, and *TFPI*) (LaFrance et al., 2015; Yuan et al., 2015; Chen et al., 2018; Luo et al., 2022) was quantified through SYBR Green-based real-time PCR procedures, following the sequences previously described in Supplementary Table S1. The reverse transcription and real-time PCR procedures were conducted as previously described methods (Xu et al., 2021). The housekeeping gene *GAPDH* was employed as an endogenous control to normalize the expression levels of the four genes (Rippe et al., 2021). The HeLa cell line was selected as a reference for further analysis of TcdB-related receptor genes expression. All experiments were independently performed in triplicate.

### 2.3 Sample collection

Clinical stool samples were collected from June to September in 2021 at the First Affiliated Hospital of Zhejiang University School of Medicine, and were stored at −80°C for subsequent detection within 48 h. Patients with diarrhea were defined as CDI when their stool samples tested positive for toxigenic *C. difficile* using the GeneXpert *C. difficile* assay combined with the TC assay (Lin et al., 2022). Individuals without diarrhea were characterized as having CDC when toxigenic *C. difficile* were detected in their stool samples using the GeneXpert *C. difficile* assay (Lin et al., 2022). Clinical information including gender, age, type of underlying medical condition, presence of diarrhea and fever, history of antimicrobial use within 8 weeks, and CDI severities determined according to the guideline (Cohen et al., 2010), was documented. The study has been approved by the Ethics Committee of Hangzhou Medical College (LL2022-01), and the requirement for informed consent was waived due to its retrospective nature.

### 2.4 *C. difficile* genotyping and preparation of purified culture supernatant

*C. difficile* isolates were obtained from clinical stool samples according to the described assay (Jin et al., 2017). Multilocus sequence typing (MLST), toxin genes *tcdA* and *tcdB*, and binary toxin genes detection was performed as previously reported (Griffiths et al., 2010; Jin et al., 2017). Data on *C. difficile* sequence types (STs) and clades were deposited in the public *C. difficile* MLST database.^1^ Isolates were cultured on Columbia Blood Agar (Thermo Fisher Scientific, Inc., Waltham, United States) for 48 h at 37°C in an anaerobic chamber with GENbag anaer (bioMérieux Inc., Durham, United States). A single *C. difficile* colony per isolate was inoculated into brain heart infusion broth, and cultured for 48 h at 37°C under anaerobic conditions. Supernatants were then collected for toxin detection using the RTCA assay, as described below.

### 2.5 CDC sample supernatants inoculated with purified TcdB

Each CDC stool sample was diluted to 20% (wt/vol) in cold PBS. Supernatants, filtered through a 0.22 μM microporous filter membrane (Merk KGaA, Darmstadt, Germany), were then pooled. The purified TcdB (2.5 ng/mL) underwent a two-fold dilution, resulting in a concentration of 1.25 ng/mL. This diluted TcdB was combined with 1 mL aliquots of the pooled CDC supernatants (CDC + TcdB) and in 1 mL of cool PBS (PBS + TcdB). For the blank control (CDC + PBS), 50 μL of pooled CDC supernatants was mixed with 50 μL of cool PBS. Prior to RTCA detection, the purified TcdB and PBS mixed with pooled CDC supernatants were individually incubated for 30 min at 37°C.

### 2.6 RTCA for detection of functional and toxical TcdB

Clinical stool samples, purified TcdB with pooled CDC supernatants, and isolated *C. difficile* culture supernatants for the RTCA assay were prepared with minor adjustments from the previously described method (Ryder et al., 2010). In brief, a 5% (wt/vol or vol/vol) stool sample was mixed in cold PBS, and supernatants were obtained after filtration through a 0.22 μM microporous filter membrane. The preparation of purified TcdB with pooled CDC supernatants and *C. difficile* culture supernatants followed the same procedure. Functional and toxical TcdB detection in stool samples, pooled CDC supernatants, or culture supernatants was conducted using the RTCA system (ACEA Biosciences Inc., San Diego, United States), following the manufacturer’s instructions, as detailed in previous reports (Ryder et al., 2010). In brief, a 96-well E-Plate was loaded with 40 μL of DMEM medium to establish baseline electrical impedance values represented by cell index (CI). Subsequently, 120 μL of HS27 cell suspension at a density of approximately 4 × 10^4^ cells/mL was added, and the E-plate with cells was placed on the RTCA SP instrument in a cell incubator with 5% CO_2_ at 37°C. CI values were automatically monitored at 10 min intervals for 16 to 18 h. After reaching a stable plateau, the CI of each sample was normalized to one at the last measurement time point before treatment. Then, 40 μL of supernatants with and without the neutralization antibody were added to the confluent HS27 cell layer. Normalized cell index (nCI) values were continuously calculated at 5 min intervals for 48 h. A stool sample was considered positive for functional and toxical TcdB when (i) the nCI value was below 0.5 and (ii) the nCI decrease was fully inhibited by a TcdB-neutralization antibody (Ryder et al., 2010). Quantitative determination of functional and toxical TcdB concentrations in stool samples, pooled CDC supernatants, or culture supernatants was performed using the established nonlinear fitting formula, correlating the time for a 50% drop in nCI with the toxin concentration, as detailed in our previous study (Ryder et al., 2010). Each sample underwent independently triplicate testing and included a positive control (40 ng/mL purified TcdB), a negative control (PBS), and a blank control (DMEM).

### 2.7 ELISA

The *C. difficile* toxin B (TCD-B) ELISA kit (Shanghai Kexing Trading Co, Ltd., Shanghai, China) was utilized as per the manufacturer’s instructions. Briefly, 10 μL of a 20% diluted stool supernatant, mixed with 40 μL sample diluent, was added to a 96-well plate well and incubated at 37°C for 10 min. After five washes with wash buffer, HRP-conjugate reagent was added to the reaction well, followed by a 30 min incubation at 37°C. Following additional washing and the addition of chromogen solution, plates were incubated at room temperature away from light for 10 min. The absorbance at 450 nm was recorded within 15 min after adding the stop solution. The calibration, constructed using the standard TcdB toxin provided by the kit, facilitated the calculation of the total TcdB protein in the original stool samples. Each test was performed in duplicate in parallel, and each sample underwent independently triplicate testing, including a positive control, a negative control, and a blank control.

### 2.8 Real-time PCR

Genomic DNAs were extracted from 200 mg or 200 μL of stool samples using the QIAamp Fast DNA Stool Mini Kit (Qiagen Inc., Hilden, Germany). The *tcdB* gene copy number was determined according to a protocol previously described (Jia et al., 2023). Quantitatively detection of *tcdB* gene copies in stool samples was performed using a BIOER LineGene 9,600 Plus PCR instrument (Bioer Technology Co., Ltd., Hangzhou, China). The *tcdB* gene copies were calculated based on a calibration curve, established by plotting cycle threshold (*C_T_*) versus gene copy number. Each sample underwent independent triplicate testing, alongside a positive control (ATCC 43255), a negative control, and a blank control.

### 2.9 Data analysis

Data analysis was conducted using SPSS version 26.0 (SPSS Inc., Chicago, United States). Differences in mRNA expression levels, TcdB concentrations, and *tcdB* gene copies between CDC and CDI samples were analyzed using *t* test or Mann–Whitney *U* tests. Results were represented as mean ± standard error of the mean (SEM). *p* < 0.05 was considered statistically significant.

## 3 Results

### 3.1 Clinical patient involved in this study

In this study, 37 hospitalized patients were involved, and their clinical information was shown in Table 1. Of them, 17 patients with CDC were asymptomatic for diarrhea, while 20 patients with CDI experienced diarrhea symptoms. No significant differences in previous antimicrobial treatments within 8 weeks were observed between patients with between CDI and CDC (*p* = 0.272). Clinical outcomes for 19 CDI cases were predominantly mild-to-moderate, with one case exhibiting severe symptoms.

**Table 1:** Clinical information of hospitalized patients with CDC and CDI in this study.

Patient characteristics	CDC (*n* = 17)	CDI (*n* = 20)
Age > 70 yrs (median, 70 yrs)	8 (47.1%)	11 (55.0%)
Gender, male, yes (*n* [%])	10 (58.8%)	15 (75.0%)
Diarrhea, yes (*n* [%])	0	20 (100.0%)
Fever, yes (*n* [%])	2 (11.8%)	3 (15.0%)
Previous antimicrobial treatment within 8 wks, yes (*n* [%])	16 (94.1%)	20 (100.0%)
Underlying disease (*n* [%])		
Tumor	3 (17.6%)	4 (20.0%)
Infection diseases	0	2 (10.0%)
Chronic diseases	14 (82.4%)	14 (70.0%)
CDI severity (*n* [%])		
None	17 (100.0%)	0
Mild or moderate	0	19 (95.0%)
Severe	0	1 (5.0%)

### 3.2 Relative mRNA expression levels of TcdB receptor-related genes

**Figure 1:**
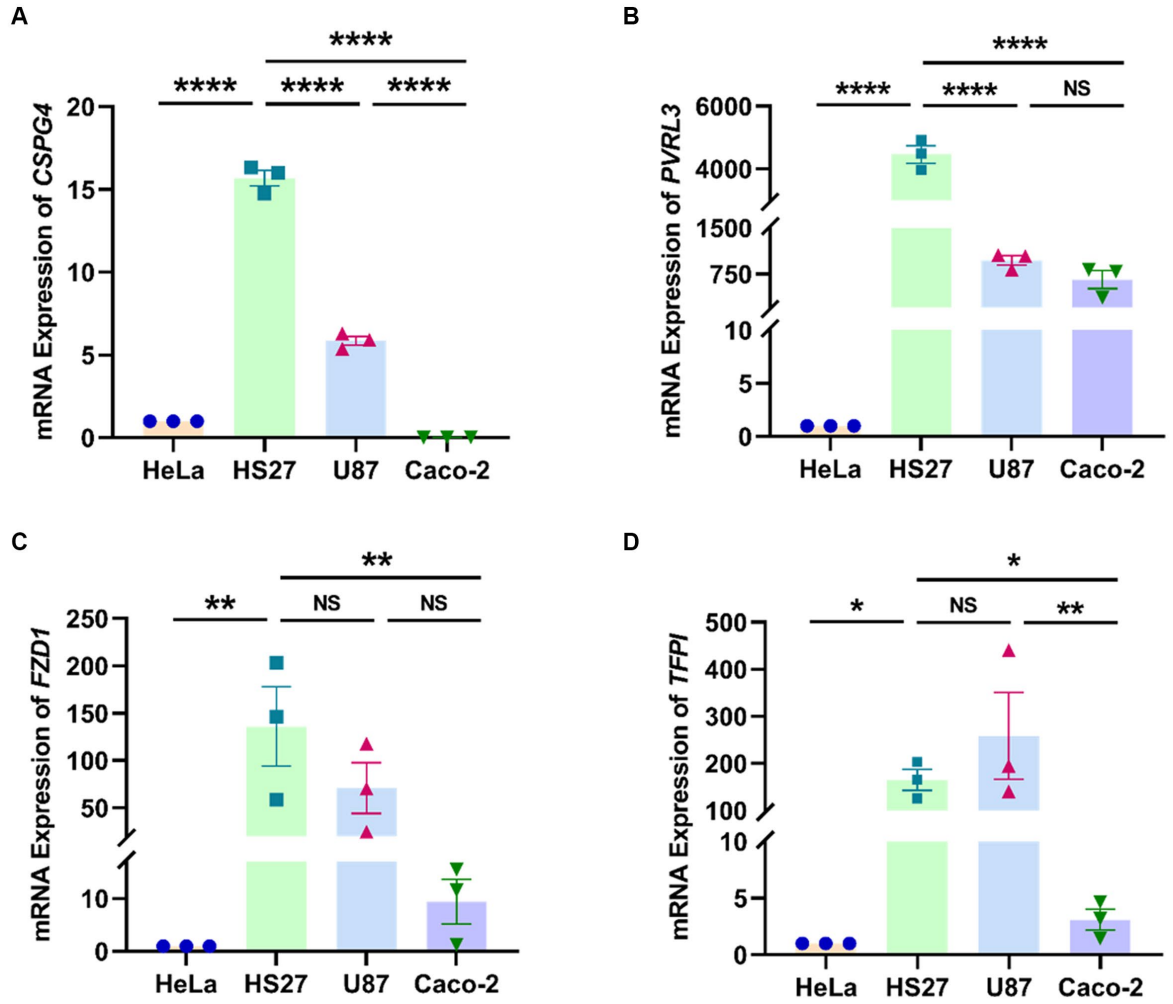
Relative expression levels of *CSPG4* **(A)**, *PVRL3* **(B)**, *FZD1* **(C)**, and *TFPI* **(D)** genes in four cell lines. ****p < 0.0001, ***p < 0.001, **p < 0.01, *p < 0.05, NS = not significant.

In the quantitative analysis of four TcdB-receptor genes (*CSPG4*, *PVRL3*, *FZD1*, and *TFPI*) across four cell lines (HeLa, HS27, U87, and Caco-2) (Figures 1A–D), we observed significantly higher relative mRNA expression levels of *CSPG4* and *PVRL3* genes in HS27 compared to U87 and Caco-2 (*CSPG4*: *p* < 0.0001; *PVRL3*: *p* < 0.0001). However, there was no significant difference in the mRNA relative expression levels of *FZD1* (*p* = 0.103) and *TFPI* (*p* = 0.200) between HS27 and U87 cells.

### 3.3 CDC and CDI samples detected using the RTCA and the ELISA

**Figure 2:**
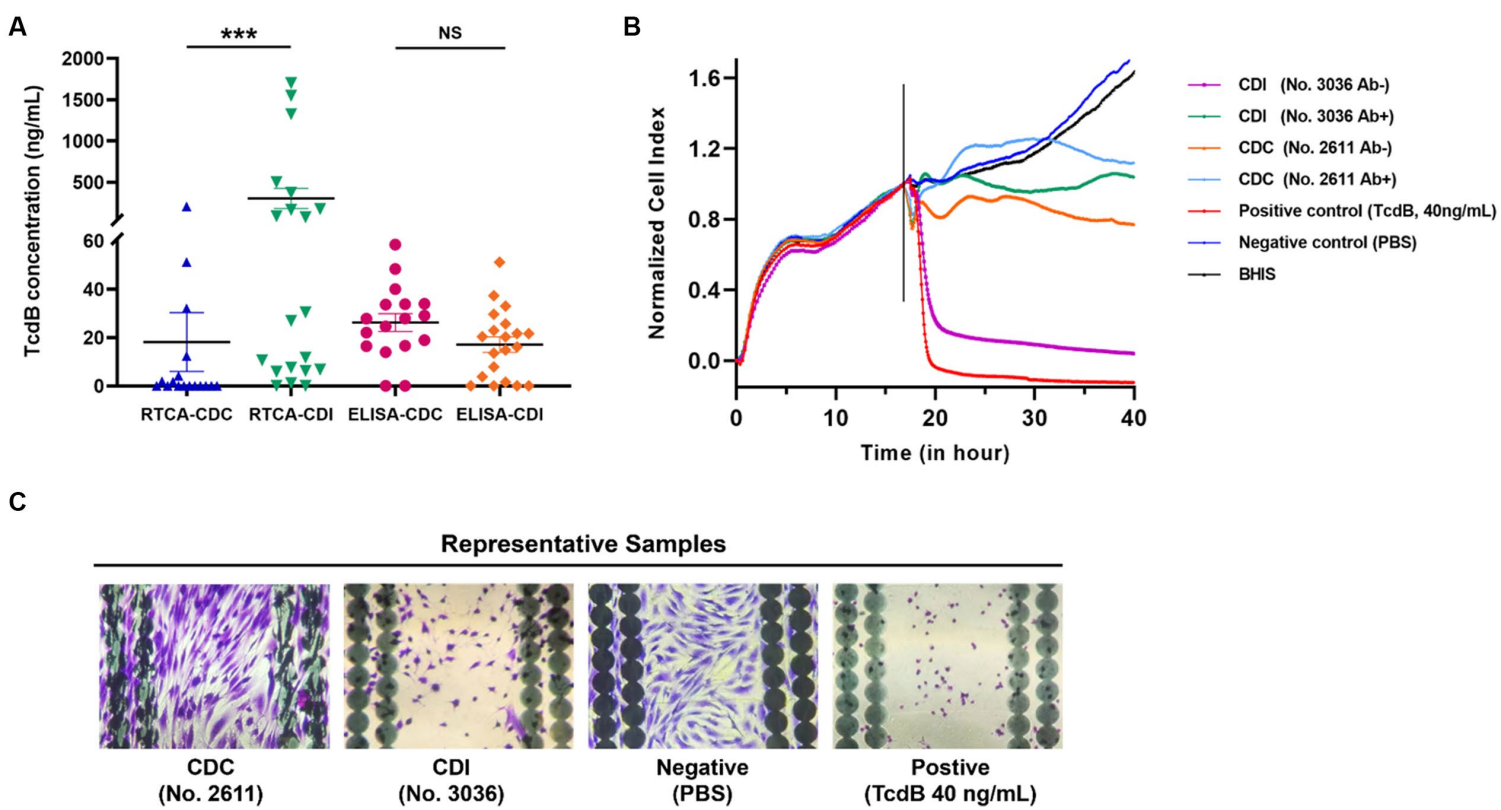
Comparative analysis of functional and toxical TcdB concentrations from CDI and CDC clinical samples, along with representative nCI curves of both were presented. **(A)** TcdB in prepared supernatants from CDI and CDC fecal samples were detected by RTCA and ELISA, respectively. Functional and toxical TcdB concentrations measured by the RTCA were calculated using the nonlinear fitting curve *y* = 7.029*x*^-0.433^. ***p < 0.001, NS, not significant. **(B)** Representative differences in cell growth curves after inoculation with CDC and CDI samples on the RTCA. The vertical lines represented the nodes of RTCA normalized analysis. Ab+: samples incubated with neutralizing antibodies; Ab−: samples incubated without neutralizing antibodies. **(C)** Stained microscopic images of cells which were grown on the E-Plate wells after CDC and CDI samples were added.

A non-linear fitting formula, utilizing purified TcdB with 7 concentrations ranging from 0.25 to 8.00 ng/mL, was developed. As shown in Supplementary Figure S1A, the observed nCI representing the cellular growth curve decreased rapidly with increasing toxin concentrations, indicating a concentration- and time-dependent TcdB-induced cytotoxicity in HS27 cells. A non-linear regression equation (*y* = 7.029*x*^-0.433^, *R*^2^ = 0.9823) was derived based on the relationship between the time for a 50% drop in nCI and known TcdB concentrations, with toxin concentration (*y*) and hours from TcdB treatment to detection (*x*) (Supplementary Figure S1B). Using this formula, functional and toxical TcdB concentrations in the 17 clinical CDC and the 20 CDI samples were quantitatively measured. After preparing stool samples as mentioned above, supernatants were divided into two aliquots. One was added into a well in the 96-E-plate, and the other was mixed with TcdB neutralizing antibody at a 1:1 ratio, subsequently incubated for 30 min at 37°C, and added into another well as described. In Figure 2A, functional and toxical TcdB concentrations measured by the RTCA system were 18.15 ± 11.81 ng/mL for the CDC group, significantly lower than the CDI group (302.58 ± 119.15 ng/mL) (*Z* = 3.256, *p* = 0.0008). However, ELISA results showed no significant differences in TcdB concentration between the CDC (26.21 ± 3.57 ng/mL) and the CDI group (17.07 ± 3.10 ng/mL) (*t* = 1.889, *p* = 0.064).

In the RTCA assay, the results displayed representative results for both CDC and CDI samples, as well as the positive and negative controls (Figures 2B,C). Ten out of 17 clinical CDC samples were negative for functional and toxical TcdB using the RTCA, with the nCI curve not dropping to below 0.5. A positive functional and toxical TcdB result was obtained from the CDI sample (No. 3036), and the time-dependent drop in nCI could be blocked by adding TcdB neutralizing antibody to the samples. However, a negative result was observed in the CDC samples (No. 2611) with no 50% drop in nCI, regardless of whether TcdB neutralizing antibody was added (Figure 2B). When the two aforementioned samples were added, the status of HS27 cells grown on the E-plate wells was checked. The results showed that this CDI sample led to a significant decrease of HS27 cells attaching the well as the positive control (purified TcdB, 40 ng/mL). However, no cytopathic effects were observed after adding this CDC sample to the well as the negative control (Figure 2C).

### 3.4 Real time PCR for detecting CDC and CDI samples

The standard *tcdB* plasmids underwent serially diluted (1.28 × 10^2^ to 2.0 × 10^6^ copies/μL), establishing a linear regression equation (*y* = −0.2901*x* + 9.3811, *R*^2^ = 0.9963) correlated with *C_T_* values (Supplementary Figure S2A). Following the detection of 37 clinical samples, *tcdB* gene copies were calculated using the standard curve. Results revealed that average *tcdB* gene copies were 774.54 ± 357.89 and 4,667.69 ± 3,069.87 copies/μL in the CDC and CDI groups, respectively (Supplementary Figure S2B). Although average *tcdB* copies in the CDI group was higher than that in the CDC group, no significant difference was observed in *tcdB* gene concentrations between the CDC and CDI samples (*Z* = 0.853, *p* = 0.407).

### 3.5 Pooled CDC sample supernatants inoculated with purified TcdB detected by the RTCA

**Figure 3:**
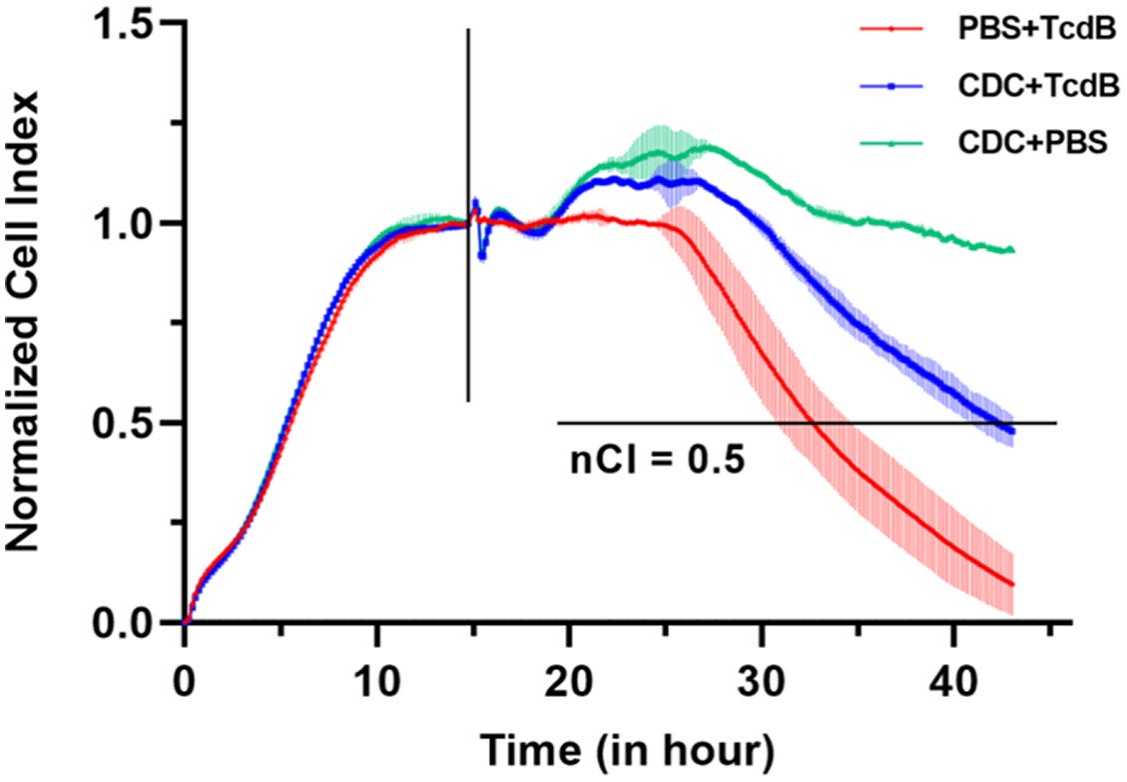
Pooled CDC supernatants inhibited TcdB activity. The vertical lines represented the nodes of RTCA normalized analysis, the horizontal line indicated the nCI 50% cutoff value.

A 40 μL of PBS + TcdB, CDC + TcdB, and CDC + PBS was added to the 96-E-Plate wells， yielding a final TcdB concentration of 0.25 ng/mL. As shown in Figure 3, RTCA cell growth curves revealed that the nCI values of the blank control did not decrease to 0.5 after 45 h of continuous monitoring. However, the time required for nCI to reach 0.5 in the CDC + TcdB group (inoculation-to-detection time: 27.48 ± 0.86 h) was significantly longer than that in the PBS + TcdB group (17.77 ± 0.94 h) (*t* = 5.68, *p* = 0.030), indicating that the concentration of functional and toxical TcdB in CDC supernatants was lower than the purified TcdB although they were the same concentration.

### 3.6 Molecular characteristics and TcdB expression of *C. difficile* isolates from clinical samples

**Figure 4:**
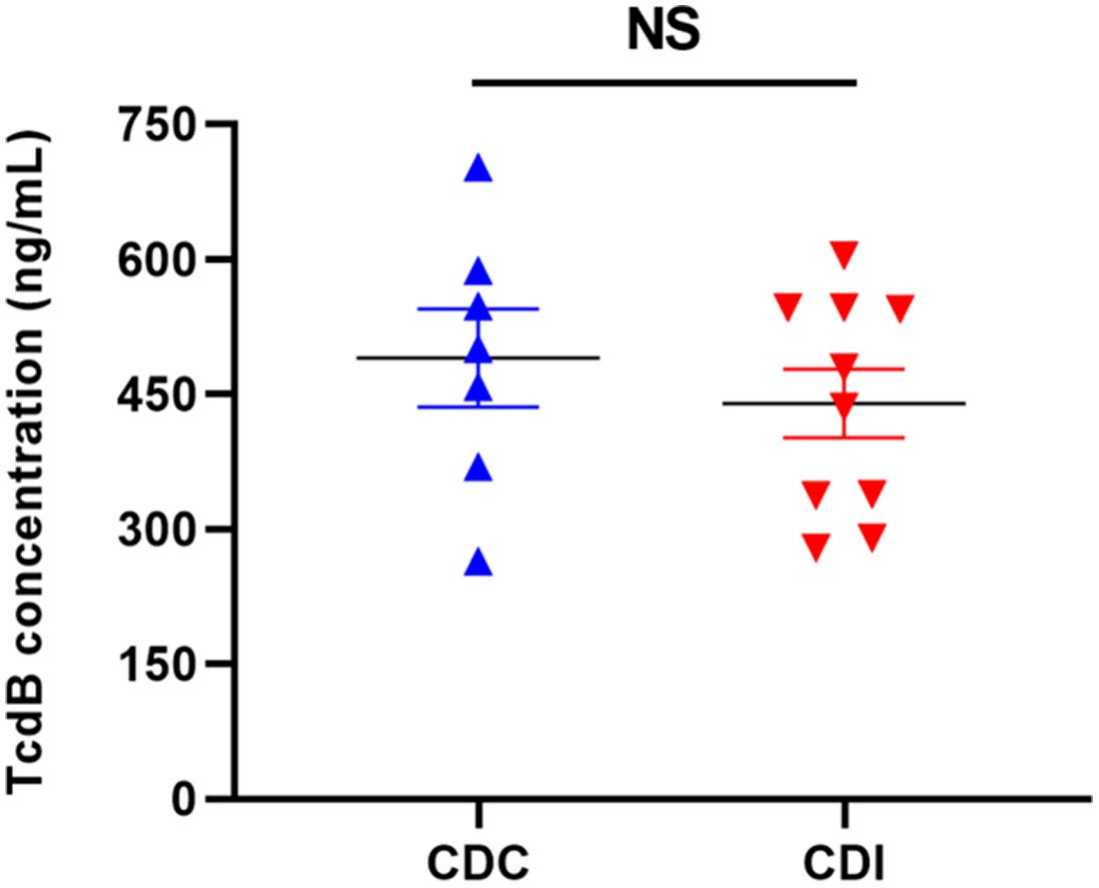
The concentrations of functional and toxical TcdB expressed by *C. difficile* isolates from CDC and CDI samples measured using the RTCA. NS, not significant.

A total of 17 *C. difficile* isolates were recovered from the 37 clinical stool samples, with 7 isolates from the CDC group and the remaining 10 from the CDI group. Results of toxin gene and genotyping are presented in Supplementary Table S2. Among the isolates, 14 (82.4%) tested positive for both *tcdA* and *tcdB*, while being negative for binary toxin genes. One isolate was positive for *tcdA*, *tcdB*, and binary toxin genes. After collecting supernatants, functional and toxical TcdB secreted from *C. difficile* isolates was measured using the RTCA assay. The results indicated that the expression levels of functional and toxical TcdB from the CDC and CDI isolates were 490.00 ± 133.29 and 439.82 ± 114.66 ng/mL, respectively, with no significant difference between the two groups (*t* = 0.780, *p* = 0.448) (Figure 4).

## 4 Discussion

It is crucial to accurately distinguish between clinical CDC and CDI for tailored clinical treatment. The RTCA system has previously been used to quantitatively detect functional and toxical TcdB in clinical stool samples, access clinical CDI severity, and evaluate antimicrobial therapy effectiveness (Ryder et al., 2010). Our findings, for the first time, highlighted the utility of RTCA in differentiating CDI and CDC through functional and toxical TcdB quantification, and further comparisons with PCR and ELISA assays underscoring its effectiveness in CDI diagnosis. Additionally, we found that CDC supernatants inhibited TcdB cytotoxicity.

TcdB, a primary virulence factors in *C. difficile*, exerts cytotoxicity by binding to specific receptors and undergoing subsequent endocytosis (Kroh et al., 2017). Despite a decade of demonstrating TcdB-induced toxicity in various cell types, the associated receptors remained elusive (Voth and Ballard, 2005). In our previous studies (Ryder et al., 2010; Huang et al., 2014a), the reasons behind the heightened susceptibility of HS27 cells to functional and toxical TcdB in the RTCA system, compared to other cells, eluded clarification. Recent confirmations of CSPG4, PVRL3, FZD1, and TFPI as receptors for TcdB have provided a foundational understanding of cellular responses to TcdB cytotoxicity (LaFrance et al., 2015; Yuan et al., 2015; Chen et al., 2018; Luo et al., 2022). In this study, we demonstrated that HS27 cells expressed *CSPG4*, *PVRL3*, *FZD1*, and *TFPI* receptor genes more prominently than the other three cell lines. Notably, U87 cells also exhibited high expression of these four receptors. However, during continuous cell growth monitoring by the RTCA, U87 cells rapidly fused and displayed time-dependent cluster growth on the E-Plate, rather than forming monolayer cells like HS27 cells. This resulted in uneven effects of TcdB cytotoxicity in each U87 cell, primarily localized to the periphery of these cell clusters, leading to inaccurate detection of the cell sensors in the RTCA system and unstable results (data not shown). These findings underscored that HS27 cells, being highly sensitive to functional and toxical TcdB, were suitable as a target cell line for detection functional and toxical TcdB using the RTCA.

Routine methods like TC, ELISA, and PCR were employed for CDI diagnosis by detection of toxin genes or proteins, and however their limitations are evident (Mah et al., 2023). While the PCR assay has high sensitivity, it cannot distinguish between CDC and CDI (Mah et al., 2023). Studies indicate that a substantial number of patients with CDI who tested PCR-positive did not exhibit clinically significant diarrhea (Kim et al., 2018), and *C_T_* values were poorly correlated with CDI outcomes (Mah et al., 2023). Due to PCR cannot identify the presence of functional and toxical toxins through detecting *tcdB* gene copies, reliance on the PCR assay alone led to the overdiagnosis of CDI (Koya et al., 2019). On the other hand, the European guideline suggests a two-step approach including PCR or GDH EIA as a preliminary screening test and a toxin A/B EIA test to diagnose CDI for enhancing specificity and sensitivity (Crobach et al., 2016). Nevertheless, some potential impact of factors such as human intestinal environment and immune status made activity of toxin proteins be inhibited (Crobach et al., 2018). Thus, this test algorithm is still unable to accurately reflect the true clinical outcome of CDI. Additionally, the TC assay is commonly conducted alongside the cytotoxic cell neutralization assay, recognized as the gold standard for CDI diagnosis; however, it is subjective, labor intensive and time-consuming, taking up to 72 h for final results (Huang et al., 2014b). The RTCA system stands out as a label-free, real-time, and non-invasive technique with high sensitivity and specificity, and it has been employed in previous studies to quantify functional and toxical TcdB concentrations in fecal sample. In our study, we found significant differences in functional and toxical TcdB concentrations when CDC and CDI samples were analyzed by the RTCA, while both ELISA and PCR failed to reveal imperative resolution capacities. These results underscored the potential of the RTCA system as an effective tool for distinguishing between CDC and CDI in clinical practice.

It has been reported that bile acids (Tam et al., 2020), short-chain fatty acids (Yuille et al., 2015), and the trace element selenium (Loureiro et al., 2022) were considered potential inhibitors of TcdB-induced cytotoxicity, however the reports on fecal TcdB inhibitors are limited. Our study showed that significant differences in functional and toxical TcdB levels were observed between patients with CDC and CDI, while their total levels of TcdB protein were comparable. Moreover, co-culturing TcdB with CDC samples resulted in a significant reduction in toxin concentration, suggesting the presence of TcdB inhibitors in CDC samples. Metabolomic analysis revealed variations in the depletion of certain bile acids between patients with CDI and CDC (Robinson et al., 2019). Additionally, bile acids primarily exhibited inhibitory effects on low to moderate TcdB levels, while high-level TcdB remained unaffected (Icho et al., 2023). This implied that bile acids might suppress TcdB within patients with CDC, explaining their absence of clinical symptoms (Icho et al., 2023). Notably, bile acids directly interacted with TcdB by binding to the C-terminal combined repetitive oligopeptides (CROPs) domain, altering TcdB conformation, and reducing its toxicity (Tam et al., 2020). However, the known receptor sites for TcdB, such as PVRL3, FZDs, TFPI, and CSPG4, are located either outside the CROPs domain or between the CROP and receptor binding domain domains (LaFrance et al., 2015; Yuan et al., 2015; Tao et al., 2016; Orrell et al., 2017; Luo et al., 2022). Therefore, there might be other inhibitors present in CDC samples. Hence, a further study has been designed to identify potential substances inhibiting TcdB cytotoxicity through comparative proteomes and metabonomics.

This study has several limitations. Firstly, the sample size was limited, and future research with a larger number of clinical stool samples was necessary to further validate the RTCA assay for detecting a broader range of *C. difficile* isolates from other clades. Additionally, we intend to further evaluate TcdB activity inhibited by CDC stool samples, and inoculate TcdB cut-off processed CDC samples into CDI samples, allowing for a comparative analysis of the concentrations of functional and toxical TcdB measured by the RTCA assay. Secondly, the efficiency of the capture antibody to enrich TcdB in clinical stools was still low. Our future studies will integrate nucleic acid aptamer technology to capture TcdB protein for simplifying detection process. Finally, the presence of functional and toxical TcdB inhibitors in CDC samples is not clearly elucidated. Subsequent studies should incorporate fecal metabolomics to determine which substance suppress the host’s inflammatory response.

In conclusion, the RTCA system proves a powerful tool for effectively distinguishing between clinical CDC and CDI through quantifying functional and toxical *C. difficile* TcdB. A large, prospective study has been designed to further evaluate the utilization of this RTCA system in standardizing antimicrobial usage and reducing economic burden in CDI treatment. Additionally, we employ a “one stone, two birds” strategy to use the RTCA system alongside host targets, such as intestinal epithelial cell lines, to identify varied unknown bacterial toxins. This involves comparing the cell line responses to curves as standards induced by known bacterial toxins, differentiating between colonization and infection led by other pathogenic bacteria.

## Data availability statement

The original contributions presented in the study are included in the article/Supplementary material, further inquiries can be directed to the corresponding authors.

## Ethics statement

The studies involving humans were approved by Ethics Committee of Hangzhou Medical College. The studies were conducted in accordance with the local legislation and institutional requirements. The ethics committee/institutional review board waived the requirement of written informed consent for participation from the participants or the participants’ legal guardians/next of kin because the written informed consent requirement was waived for this study involving ex vivo fecal samples due to the nature of the research, which focuses exclusively on anonymized and de-identified specimens. The study poses minimal risk to participants as it does not involve any personal or sensitive information. Waiving written informed consent was deemed appropriate and in compliance with ethical standards to ensure participant privacy while conducting a scientifically rigorous investigation.

## Author contributions

YS: Methodology, Writing – original draft. SL: Methodology, Writing – original draft. PY: Data curation, Formal analysis, Writing – original draft. YuC: Formal analysis, Writing – original draft. YL: Formal analysis, Writing – original draft, Writing – review & editing. XS: Investigation, Methodology, Writing – original draft, Writing – review & editing. YunC: Conceptualization, Writing – review & editing. DJ: Conceptualization, Writing – original draft, Writing – review & editing, Funding acquisition, Supervision.

## Footnotes

^1^ https://pubmlst.org/cdifficile/
