# Supplementary material for: Rapid discrimination between clinical Clostridioides difficile infection and colonization by quantitative detection of TcdB toxin using a real-time cell analysis system
Source: Front Microbiol. 2024 Jan 23;15:1348892. doi: 10.3389/fmicb.2024.1348892 (PMC10844495; doi:10.3389/fmicb.2024.1348892)
Supplement: Supplementary file 1 [file Data_Sheet_1.docx]

Supplementary Material

# Supplementary Tables

## Table S1 Nucleotide sequences of the oligonucleotide primers used in the study

| **Target** | **Primer** | **Oligonucleotide sequence (5' - 3')** |
| --- | --- | --- |
| *CSPG4* | mCSPG4-RT-F | TCTACCGGGTGGTAAGAGGC |
|  | mCSPG4-RT-R | TTCCCAGCATTTACCTCAGCC |
| *FZD1* | mFZD1-RT-F | CATCGAGGCCAACTCACAGT |
|  | mFZD1-RT-R | TGAGCCCCACAAAACACACT |
| *PVRL3* | mPVRL3-RT-F | ATTGGTTTGTAGGAAGAAAAGGTGT |
|  | mPVRL3-RT-R | AAATGAAGAGTATTGTCTGAAGCCA |
| *TFPI* | mTFPI-RT-F | GCAGCATCTGGAGCAGAAAG |
|  | mTFPI-RT-R | TGTAACCTCGGCAGAGTCCA |
| *GAPDH* | mGAPDH-RT-F | ATCCTGCACCACCAACTGCT |
|  | mGAPDH-RT-R | GGGCCATCCACAGTCTTCTG |

## Table S2 Toxin genes, MLST and clade of *C. difficile* isolates from CDI and CDC stool samples

| **Serial number** | **Strain name** | **Toxin genes** | **MLST** | **Clade** |
| --- | --- | --- | --- | --- |
| 1 | CDC 0401 | A^-^B^-^CDT^-^ | 48 | 1 |
| 2 | CDC 0412 | A^+^B^+^CDT^-^ | 54 | 1 |
| 3 | CDC 1019 | A^+^B^+^CDT^-^ | 4 | 1 |
| 4 | CDC 1510 | A^-^B^-^CDT^-^ | 48 | 1 |
| 5 | CDC 1701 | A^+^B^+^CDT^-^ | 98 | 1 |
| 6 | CDC 2420 | A^+^B^+^CDT^-^ | 42 | 1 |
| 7 | CDC 2711 | A^+^B^+^CDT^-^ | 2 | 1 |
| 8 | CDI 0604 | A^+^B^+^CDT^-^ | 54 | 1 |
| 9 | CDI 0607 | A^+^B^+^CDT^-^ | 129 | 1 |
| 10 | CDI 0710 | A^+^B^+^CDT^-^ | 54 | 1 |
| 11 | CDI 1705 | A^+^B^+^CDT^-^ | 2 | 1 |
| 12 | CDI 1932 | A^+^B^+^CDT^+^ | 11 | 5 |
| 13 | CDI 2409 | A^-^B^+^CDT^-^ | 37 | 4 |
| 14 | CDI 2603 | A^+^B^+^CDT^-^ | 2 | 1 |
| 15 | CDI 2807 | A^+^B^+^CDT^-^ | 2 | 1 |
| 16 | CDI 3002 | A^+^B^+^CDT^-^ | 2 | 1 |
| 17 | CDI 3036 | A^+^B^+^CDT^-^ | 233 | 1 |

# Supplementary Figures

**Fig. S1.** Dynamic responses of HS27 cells reduced by the purified TcdB for establishing a non-linear fitting formula.

**
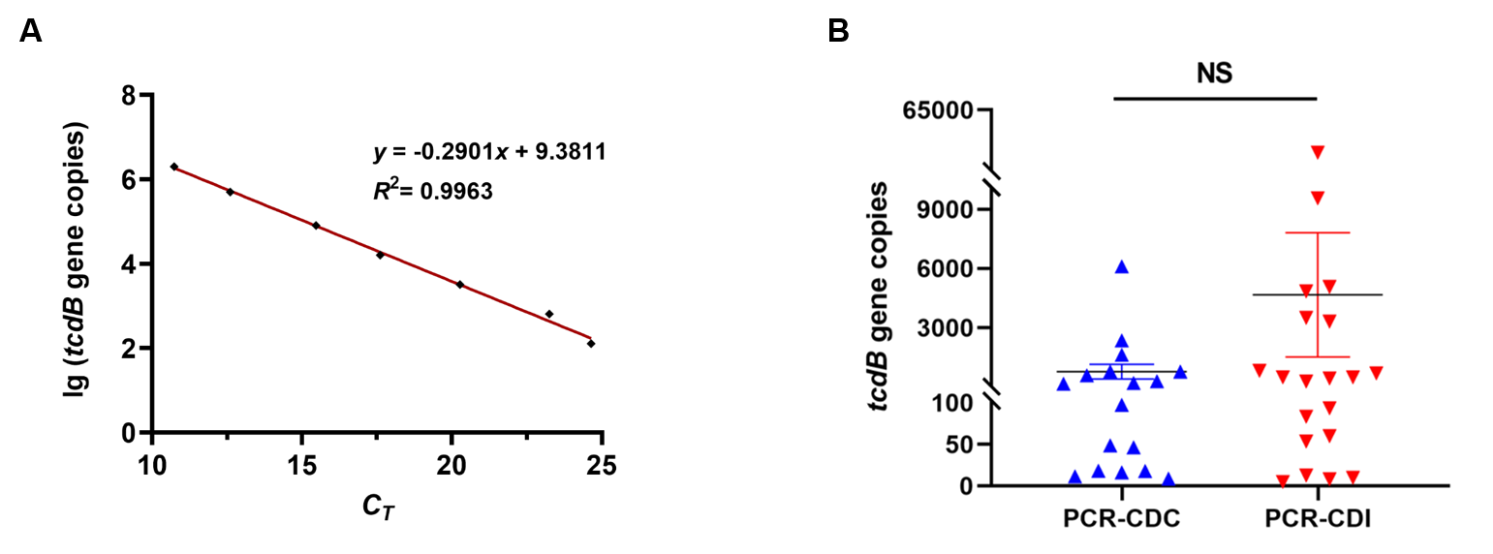
**

**Fig. S2.** CDC and CDI samples detected by the real-time PCR.
